# Errata

**Published:** 1782

**Authors:** 


					f. 211
A ^p, for L&Ker reait'uwtr i 2?4V A I, Jor hepatide read hepatuwie

				

